# The Influence of Clinical and Environmental Risk Factors in the Etiology of Congenital Sensorineural Hearing Loss in the Romanian Population

**Published:** 2019-12

**Authors:** Horia MOCANU, Ionica ONCIOIU

**Affiliations:** Faculty of Medicine, Titu Maiorescu University, Bucharest, Romania

## Dear Editor-in-Chief

“The integrity of the auditory system is one of the prerequisites for the acquisition and the proper development of oral language since it is through interaction with others that children acquire language, understand their universe, their peers, develop and organize thoughts and feelings, and gain knowledge (1)”. Congenital hearing loss is relatively frequent, with a prevalence reported by different sources in literature as varying from 1–3/1000 newborns (NBs) to 1/500 newborns (2, 3). Recent discoveries indicate the mutations of the GJB2 and GJB6 genes on the 13q11–q12 chromo-some as responsible of more than 50% of all types of non-syndromic autosomal recessive congenital hearing loss in certain populations (4). Although etiologically heterogeneous, at least 50% of all early on-set hearing losses have a genetic cause while the rest is directly linked to the presence of environmental and perinatal risk factors (5).

Romania, as an East-European developing country has, unfortunately, no coherent strategy for new born SNHL diagnosis which should comprise of, at least, screening procedures such as Transient Evoked Otoacoustic Emissions (TEOAE) and evaluation of known risk factors, not to mention the follow-up of all infants who present such indicators. Most maternities in the country do not perform any kind of hearing screening for new-borns as recommended in the literature (6, 7). Thus, most of the congenital sensorineural hearing loss (SNHL) cases are diagnosed after the age of 3 and this becomes a matter of public health concern due to the serious impairment on language development and social integration (8).

We tried to evaluate the frequency of environmental and perinatal risk factors and their statistical significance on the occurrence of congenital SNHL in the new born population of one of the busiest maternities Children’s Clinical Emergency Hospital “Marie Sklodowska Curie”, in Bucharest, Romania, from October 2017 to April 2018.

In this cross-sectional and retrospective cohort study, from 58 cases with severe or profound bilateral SNHL, only 34 cases (20 female and 14 male), ages between 2 and 10 (mean age 4.62 yr), were selected. Children that had no other health issues (non-syndromic cases) and had no family history of SNHL (normal hearing parents). The demographic and clinical parameters (via a special questionnaire and the newborn’s family and clinical history) were analyzed and statistically correlated to the result of the hearing screening in order to assess the influence of risk factors on the occurrence of congenital SNHL. The initial cohort was also stratified in at term born (TB) and premature (PM) children and statistical correlations were calculated for both situations.

According to Table 1, 29.4% of cases (10/34) were homozygotic for the 35 delG mutation (35delG/35delG), also known as genotype Δ/Δ (the Greek letter Δ preceding the name of a gene, signifies that the gene has a chromosome deletion whilst the letter N represents the lack of a deletion). No case of heterozygosity 35delG/N (Δ/N) was present for the 35delG mutation whilst 5.88% of cases (2/34) belong to the heterozygotes bi-genic group 35delG/W24X.The W24X mutation was present in 5.88% of cases (2/34) as homozygotes genotype (W24X/W24X) and in 2.94% of cases (1/34) as heterozygotic genotype (W24X/N).

**Table 1:** Prevalence (%) of genotypes in the present study compared to other published studies from Romania

***Genotype***	***Present study***	***Neagu 2013 (8)***	***Totolin 2011 (7)***	***Lazăr 2010 (6)***
35delG/	10/34 (29.4)	30/84 (36)	6/26 (24)	19/75 (25.33)
35delG				
35delG/N	0/34 (0)	15/84 (18)	5/26 (19)	0/75 (0)
N/N	19/34 (55.88)	39/84 (46)	15/26 (57)	-
W24X/W24X	2/34 (5.88)	N.A.	N.A.	1/75 (1.33)
W24X/N	1/34 (2.94)	N.A.	N.A.	-
35delG/W24X	2/34 (5.88)	N.A.	N.A.	2/75 (2.66%)

N.A. – Not analyzed

The etiology of congenital SNHL entails environmental, clinical and genetic factors. Hearing disorders also were significantly associated with the presence of maternal pathologies but not with other prenatal or environmental factors such as radiation, illegal drugs etc. For the term born group, the statistically significant risk indicators were: congenital infections, ototoxic medication, neonatal hypoxia, mechanical ventilation, Neonatal Intensive Care Unit (NICU) admittance, Hypoxic Ischemic Encephalopathy Perinatal (HIEP) and hyperbilirubinemia. In the premature group the most significant risk indicators were related to the prematurity itself: gestational age, birth weight (very low birth weight, extremely low birth weight), Apgar score, respiratory distress, hypoxia, mechanical ventilation, NICU admittance, ototoxic medication and other abnormalities.

Some risk factors (prematurity, birth weight, mechanical ventilation, ototoxic medication etc.) were statistically significant in the occurrence of hearing loss and are recognized as such in literature. Other risk factors may also play an important role in this pathology.
